# 松花粉干预卵巢早衰大鼠肝脏的广泛靶向代谢组学分析

**DOI:** 10.3724/SP.J.1123.2022.04017

**Published:** 2023-01-08

**Authors:** Tao QU, Yang CHEN, Changjun YANG, Qisong LIU, Hui CHEN, Zhiyong HE, Zhaojun WANG, Jie CHEN, Maomao ZENG

**Affiliations:** 1.江南大学, 食品科学与技术国家重点实验室, 江苏 无锡 214122; 1. State Key Laboratory of Food Science and Technology, Jiangnan University, Wuxi 214122, China; 2.烟台新时代健康产业有限公司, 山东 烟台 264006; 2. New Era Health Industry (Group) Co., Ltd., Yantai 264006, China

**Keywords:** 超高效液相色谱-三重四极杆质谱, 广泛靶向代谢组学, 维生素, 松花粉, 卵巢早衰, 肝脏, ultra-high performance liquid chromatography-triple quadrupole mass spectrometry (UHPLC-MS/MS), pseudotargeted metabolomics, vitamins, pine pollen, premature ovarian failure, liver

## Abstract

卵巢早衰是妇科领域的常见病,中医认为卵巢早衰与肝肾的正常与否息息相关,通过药食两用物质对肝脏代谢的调理是治疗卵巢早衰的一种重要手段。该研究基于超高效液相色谱-三重四极杆质谱(UHPLC-MS/MS)建立的广泛靶向代谢组学技术,探讨破壁松花粉对环磷酰胺诱导的卵巢早衰模型大鼠肝脏代谢的影响,旨在通过测定对照组、模型组、雌激素阳性对照组及施以不同剂量的松花粉干预组的SD大鼠肝脏组织中代谢物的含量变化,结合主成分分析(PCA)、正交偏最小二乘判别分析(OPLS-DA)等多元统计方法揭示松花粉干预卵巢早衰大鼠肝脏代谢的作用机制。通过正、负离子模式共检测出687种肝脏代谢物,PCA与OPLS-DA显示环磷酰胺诱导的模型组能够与对照组、阳性对照组、松花粉干预组等组别之间的代谢物较好的分离。通过单变量分析中的*t*检验(*p*<0.05)、变异倍数(FC>2或<0.5)与多变量分析中变量投影重要性(VIP值)>1相结合对差异代谢物进行筛选。与对照组相比,模型组SD大鼠肝脏中的32个生物标志物含量显著升高,28个生物标志物含量显著降低,主要涉及*α*-亚麻酸代谢、维生素B6代谢、嘌呤代谢、赖氨酸降解和糖酵解/糖异生途径;松花粉的干预可以使代谢物回调,以雌激素组为参考,松花粉的有效干预主要涉及维生素B6的代谢途径。研究表明,花粉提取物可以调节伴随卵巢早衰的肝脏代谢紊乱,促进代谢向正常水平回归。

## Abstract (en)

Premature ovarian failure (POF) is a prevalent gynecological disease. In traditional Chinese medicine, it is believed that POF is directly related to abnormal function of the liver and kidneys. As such, regulation of the liver metabolism through the use of medicinal and edible substances is important for the treatment of POF. Pine pollen, a traditional Chinese medicinal and edible pollen variety, contains various active substances, such as sex hormones and phytohormones, which have been used to inhibit inflammation, regulate the immune system, and protect reproductive tissues. Using ultra-high performance liquid chromatography-triple quadrupole mass spectrometry (UHPLC-MS/MS), this study examined the influence of pine pollen on the liver metabolome of cyclophosphamide-induced POF model Sprague Dawley (SD) rats. The variations in the metabolites present in the liver tissue of control SD rats, model SD rats, and SD rats treated with various doses of pine pollen or estrogen were analyzed using principal component analysis (PCA) in combination with orthogonal partial least squares discriminant analysis (OPLS-DA) and other multivariate statistical methods to reveal the mechanism of pine pollen intervention in the livers of POF SD rats.

An animal model experiment was conducted using six groups of ten-week-old rats. Cyclophosphamide was administered intraperitoneally to the model group and four intervention groups at a dosage of 60 mg/kg for 1 d followed by a dosage of 10 mg/kg for 14 d. Within the following four weeks, each of the four intervention groups received the intragastric administration of 0.1, 0.5, or 1.5 g/kg bodyweight (BW) of pine pollen, or 0.075 g/kg BW of conjugated estrogens (positive control). Equal quantities of normal saline were administered to the control and cyclophosphamide-treated model groups. Subsequently, the rat livers were subject to pseudotargeted metabolomics, and a total of 687 liver metabolites were discovered using both positive and negative ions. The metabolites differing in content were screened using the *t*-test (*p*<0.05) and the fold change (FC>2 or <0.5) in univariate analysis, and the variable importance in projection (VIP>1) in multivariate analysis. It was found that in comparison with the control group, the contents of 32 metabolites significantly increased, while those of 28 metabolites significantly decreased in the model group. The majority of these metabolites were involved *α*-linolenic acid metabolism, vitamin B6 metabolism, and purine metabolism, along with the lysine degradation and glycolysis/gluconeogenesis metabolic pathways. Compared with the cyclophosphamide-induced model group, the estrogen group exhibited increased levels of 47 metabolites and decreased levels of 29 metabolites, wherein 34 metabolites were restored to the levels found in the control group. These metabolites mainly involved the vitamin B6, lysine, glycolysis/gluconeogenesis, arginine and proline, and cysteine and methionine metabolic pathways. In the low/medium/high-dose pine pollen groups, the contents of 34/32/34 metabolites increased, the contents of 30/37/24 metabolites decreased, and the contents of 47/38/34 metabolites were restored to the levels found in the control group, respectively. These metabolites were mainly involved in vitamin B6 metabolism, purine metabolism, and the glycolysis/gluconeogenesis metabolic pathway. These results therefore indicate that the restoring effect of pine pollen is equivalent or superior to that of conjugated estrogen. Additionally, based on the known metabolic pathways, it appears that when estrogen interferes with the liver metabolism, the key metabolic pathways that become affected are the arginine and proline metabolism and cysteine and methionine metabolism pathways. In contrast, pine pollen intervention affected existing metabolic pathways that were known to be disordered by cyclophosphamide. The use of pine pollen may therefore restore the levels of many metabolites. It should be noted that 23 overlaps exist between the estrogen-restored metabolites and the pine pollen-restored metabolites, including a variety of acylcarnitines, such as ACar 10∶0. As a result, pine pollen extract may be able to normalize the liver metabolic abnormalities induced by POF. This study therefore establishes a theoretical reference for the development of functional applications for pine pollen and for the treatment of POF.

卵巢早衰是妇科领域的常见病,通常伴随着闭经、雌激素不足、促性腺激素水平升高和不孕症。中医认为,肾为先天之本,经水的正常与否与肾密切相关,肾的盛衰之变化决定了月经能否正常潮至,而肝脏与肾的联系密切,李中梓在《医宗必读》中指出肝肾本为同源,“补肾即补肝”^[1]^,因此存在从肝论治卵巢早衰的观点^[2]^。西医认为,卵巢早衰的病因广泛,可能由染色体缺陷等遗传性因素、化疗等医源性因素以及环境因素等影响造成。肝脏可在碳水化合物、脂质、蛋白质和微量元素代谢中发挥重要作用,因此卵巢早衰伴有肝脏功能和代谢层面的改变不足为奇。^[3]^,此外Yomna^[4]^在其研究中更是证实了慢性胆汁淤积这一肝病会导致雌性大鼠性腺功能减退和卵巢早衰。

环磷酰胺是常用的化疗药物和免疫抑制药物之一,但其使用过程中对女性卵巢的毒性反应强,易引起卵巢损伤和早衰。实验表明,环磷酰胺处理后大鼠的卵巢萎缩,卵泡数减少,雌激素水平下降,促卵泡素升高,与临床卵巢早衰患者血清学表现一致,可为卵巢功能损伤的发病机制及药物保护机制的探索提供有效的动物模型^[5]^。环磷酰胺在体内须经过肝脏细胞色素P450酶代谢后才能发挥作用,但其代谢产物如丙烯醛^[6]^或超氧阴离子、过氧化氢等活性氧(reactive oxygen species, ROS)^[7]^会抑制肝脏的抗氧化防御机制,从而诱导肝损伤。

无论是出于通过从肝论治卵巢早衰^[1]^,还是抑制环磷酰胺的肝损伤副作用^[8]^,以往的研究中都有将天然抗氧化剂的摄入当作重要的干预治疗手段的报道。松花粉又称松黄,是我国松科植物马尾松(*Pinusmassoniana* Lamb.)、油松(*Pinustabuliformis* Carr.)及同属种植物的雄性生殖细胞,主要营养成分包括蛋白质、氨基酸、维生素等,是我国传统药食两用的花粉品种^[9]^。最新研究显示,松花粉中含有雌二醇(E2)、睾酮(T)及孕酮(P)3种性激素、赤霉素类植物激素^[10]^和多种结合态的酚类化合物^[9]^等多种天然活性物质,因而在抑制炎症反应^[11]^、调节免疫^[12]^、延缓睾丸组织衰老^[13]^和肝损伤保护^[14]^等方面都有一定的效果。因此,通过研究松花粉干预对卵巢早衰动物模型中肝脏代谢的影响对卵巢早衰及其干预的机制研究均有重要意义,但目前关于松花粉干预卵巢早衰伴随肝脏表型改变的代谢组学研究尚未见报道。

代谢组学的任务在于研究机体应对精神压力和疾病等情况时所引起机体内源性小分子代谢物的变化用于理解生物学机制,目前多通过液相色谱-质谱联用、气相色谱-质谱联用和核磁共振3种技术平台实现,因液相色谱-质谱联用技术具有灵敏度高、分辨率高,可分析不稳定、不易衍生化和难挥发代谢物等优点成为代谢组学研究中常用的分析平台^[15]^。非靶向代谢组学不需要化学标准品,旨在无偏向性地检测尽可能多的代谢物,通常具有卓越的代谢物覆盖率以及简单的方法开发过程,然而,它在化合物鉴定和量化方面表现不佳。靶向代谢组学使用化学标准品来关注有限的已知代谢物,表现出卓越的定量性能,但涉及复杂的方法开发,并且具有较差的代谢物覆盖率。广泛靶向代谢组学又称拟靶向代谢组学,被认为是第二代代谢组学方法,它融合了靶向和非靶向代谢组学数据的采集策略,实现兼具高覆盖度和高性能的定量分析^[16]^,具有代谢物特征离子对覆盖度广、灵敏度高、稳定性好等特征。

本文首先采用环磷酰胺诱导雌性SD大鼠建立卵巢早衰模型,进一步采用基于超高效液相色谱-三重四极杆质谱 (UHPLC-MS/MS)的广泛靶向代谢组学研究了不同剂量松花粉摄入对卵巢早衰大鼠肝脏代谢的干预情况,并以雌激素干预组作为阳性对照,结合单变量分析与多变量分析来确定环磷酰胺诱导卵巢早衰模型时与正常组大鼠相比的肝脏差异代谢物,从而以此为参考确定雌激素和不同剂量松花粉对模型组大鼠肝脏代谢物的有效回调。将雌激素组和松花粉组共同的有效回调差异代谢物进行代谢通路分析,并作为松花粉可以与雌激素发挥一致作用的干预机制。本研究对研究卵巢早衰的代谢机制及干预方法提供了理论和实验依据。

## 1 实验部分

### 1.1 仪器、试剂与材料

选用浙江维通利华实验动物技术有限公司SPF级10周龄的雌性SD大鼠,饲养于江南大学动物实验中心,动物实验方案经江南大学实验伦理委员会批准,审批号:JN. No20201030S1201203[272],所有动物实验操作符合江南大学动物管理与使用委员会的规定(SYXK 2012-0002)。

破壁松花粉,烟台新时代健康产业有限公司;环磷酰胺一水合物,上海百灵威化学技术有限公司;生理盐水,四川科伦药业股份有限公司;红丽来结合雌激素片,新疆新姿源生物制药有限责任公司,批号为20200404;色谱纯甲醇,德国Merck公司;内标:肉碱C2∶0-d_3_、肉碱C8∶0-d_3_、肉碱C10∶0-d_3_、软脂酸-16,16,16-d_3_、硬脂酸-18,18,18-d_3_、苯丙氨酸-d_5_、色氨酸-d_5_和鹅去氧胆酸-d_4_,美国Sigma-Aldrich公司。

SCIENTZ-48高通量组织研磨器(宁波新芝生物科技股份有限公司); CentriVap真空离心浓缩仪(美国Labconco公司); Milli-Q超纯水仪(美国默克公司); ExionLC AD型超高效快速液相色谱(美国AB SCIEX公司); Triple Quad 5500三重四极杆复合线性离子阱液相色谱-质谱联用仪(美国AB SCIEX公司)。

### 1.2 实验条件

#### 1.2.1 动物造模及分组

SPF级健康雌性SD大鼠36只(10周龄,体重(250±20) g),饲养于屏障环境中(无特定病原体,温度22~24 ℃,湿度55%~70%, 12 h昼夜交替),饲养期间大鼠自由饮食饮水。经过一周适应性喂养后随机分为6组,分别为空白对照组、环磷酰胺模型组、雌激素阳性对照组(0.075 g/kg)、松花粉低剂量组(0.1 g/kg)、松花粉中剂量组(0.5 g/kg)、松花粉高剂量组(1.5 g/kg)。除空白对照组外的30只大鼠,腹腔注射环磷酰胺(60 mg/kg)1天+环磷酰胺(10 mg/kg)14天,建立诱导性卵巢早衰模型。松花粉溶于生理盐水中制成混悬液,采用灌胃方式给药,阳性对照组灌胃结合雌激素溶液,空白对照组和环磷酰胺模型组大鼠则灌胃等体积的生理盐水,给药频率为每天一次,共4周。

#### 1.2.2 代谢物的提取与分析

精密称取肝脏组织样本约50.0 mg于2 mL离心管中。精密加入5倍体积含有脂肪酸0.5 μg/mL C16∶0-d_3_和0.5 μg/mL C18∶0-d_3_,肉碱0.16 μg/mL C2∶0-d_3_、0.1 μg/mL C8∶0-d_3_和0.1 μg/mL C10∶0-d_3_,氨基酸3.61 μg/mL苯丙氨酸-d_5_和4.25 μg/mL色氨酸-d_5_, 含0.3 μg/mL鹅去氧胆酸-d_4_的75%甲醇溶液。向离心管中加入2粒钢珠,在4 ℃条件下以66 Hz频率研磨4 min。在4 ℃条件下以11000 r/min的转速离心15 min。吸取全部上清液于新的2 mL离心管中,进行冻干处理,待样本冻干后置于-20 ℃冰箱中保存备用。在进行上机分析前,将冻干后的提取液样本,用100 μL 20%甲醇水溶液复溶。将各组待分析的样品取等体积上清液,混合,涡旋振荡,制成质控(quality control, QC)样本。

正离子模式分析条件:Waters Acquity BEH C8色谱柱(100 mm×2.1 mm, 1.7 μm),柱温为50 ℃;流动相A为0.1%甲酸水溶液,B为 0.1%甲酸乙腈溶液;线性梯度程序:0~1 min,95%A;1~24 min,95%A~0A;24~28 min,0A;28~28.1 min,0A~95%A;28.1~30 min,95%A。流速0.35 mL/min。喷雾电压设置为5.5 kV,碰撞电压设置为15、30和45 V。扫描范围设置为50~1250 Da。

负离子模式分析条件:Waters Acquity HSS T3色谱柱(100 mm×2.1 mm, 1.8 μm),柱温为55 ℃;流动相C为6.5 mmol/L NH_4_HCO_3_水溶液,D为6.5 mmol/L NH_4_HCO_3_ 95%甲醇水溶液;线性梯度程序: 0~1 min,98%C;1~18 min,98%C~0C;18~22 min,0C;22~22.1 min,0C~98%C;22.1~25 min,98%C。流速0.35 mL/min。喷雾电压设置为-4.5 kV,碰撞电压设置为-15、-30和-45 V。扫描范围设置为50~1250 Da。

### 1.3 数据处理

各分组均有6个生物学重复以筛选稳定的差异代谢物。代谢物的原始质谱数据在SCIEX OS软件(美国AB SCIEX公司)上进行处理,导出各组代谢物数据后使用MetaboAnalyst 5. 0 (https://www.metaboanalyst.ca/MetaboAnalyst/home.xhtml)对各组样本采用主成分分析(PCA)、正交偏最小二乘判别分析(OPLS-DA)、单变量分析和差异代谢物所涉及的代谢通路分析。

## 2 结果与讨论

通过正负离子模式共检测出687种物质,其中正离子模式检测出371种,负离子模式检测出316种。PCA通过不对样品加以分组的情况下进行数据分析,反映了数据的原始状态,因此可以通过PCA分析观察不同组间的差异。如图1和图2所示,QC样本在PCA图中聚集度高,表明本实验所用的仪器及数据采集稳定性及重复性较好。

**图1:**
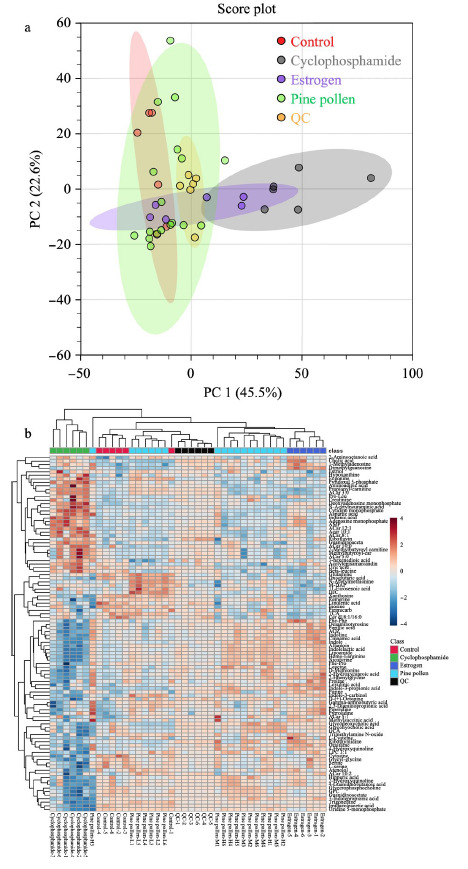
正离子模式下大鼠肝脏代谢物的PCA得分图及热图

**图2:**
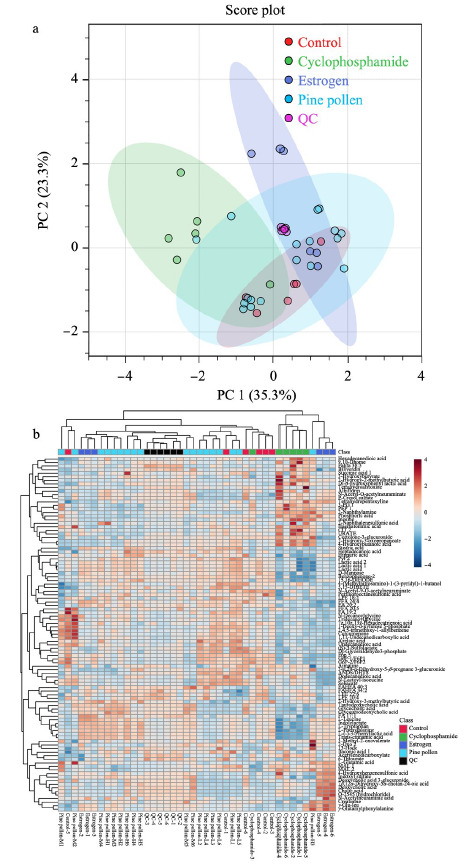
负离子模式下大鼠肝脏代谢物的PCA得分图及热图

正离子模式PCA得分图显示第一和第二主成分可以解释原始数据集68.1%的特征,在第一主成分方向上可以发现,尽管环磷酰胺模型组与空白对照组及其余组有部分交叉但分离趋势明显。聚类热图反映了代谢物的积累模式差异,结果显示,样本分组聚类一共分为了2大簇,环磷酰胺组与其余组别代谢物有明显差异,不同的生物学重复之间也同样聚成一簇,表明生物学重复之间良好的同质性和数据的高可靠性。负离子模式PCA结果(见图2)也显示每组的6个样本可以聚类在95%的置信区间内,说明各组样本的特征相似,实验数据稳定可靠。聚类热图中环磷酰胺组也基本聚成一簇。总的来说,聚类分析和主成分分析结果都可以表明本实验建模成功,环磷酰胺模型组的代谢物与其余组别的代谢物产生了明显的差异,满足探究松花粉干预卵巢早衰大鼠肝脏代谢的条件。

为反映不同组别之间的差异关系,更好地对模型进行解释,进一步采用OPLS-DA对空白对照组与环磷酰胺组,雌激素与环磷酰胺组,低、中、高松花粉组与环磷酰胺组之间进行两两成对的分析并绘制得分图。在OPLS-DA模型中,*R*^2^*Y*表示所建模型对Y矩阵的解释率,*Q*^2^表示模型的预测能力。如图3和图4所示,不同分组样本间在OPLS-DA图上能够完全分离,*R*^2^*Y*的范围在0.755~0.946之间,*Q*^2^的范围在0.578~0.911之间,可见OPLS-DA模型稳定可靠,模型的拟合度和预测能力较好,可以进行下一步差异代谢物的寻找。

**图3:**
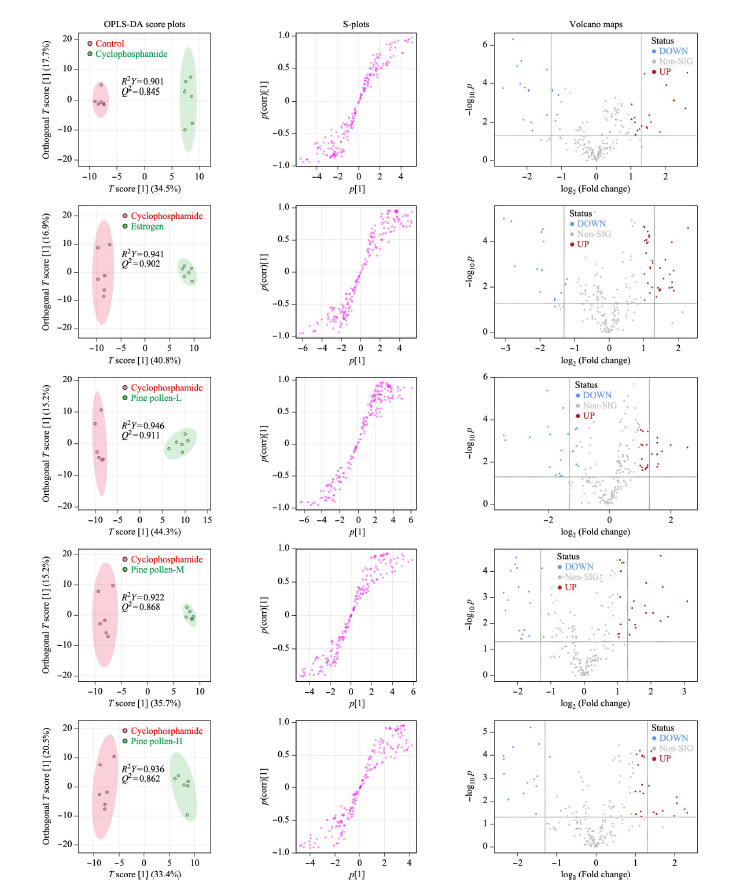
正离子模式下各组的OPLS-DA得分图、S-plot图及火山图

**图4:**
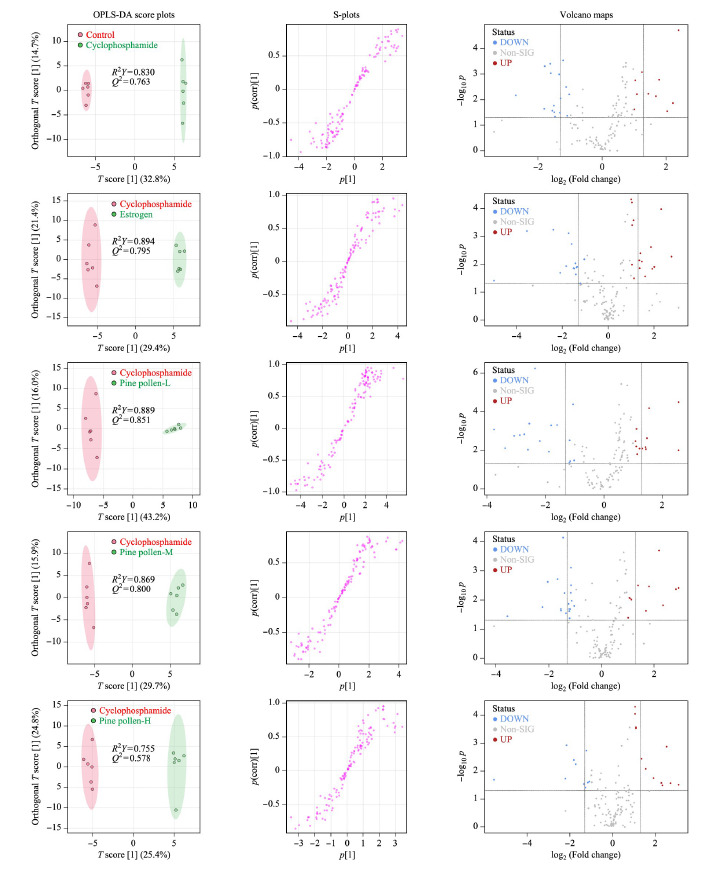
负离子模式下各组的OPLS-DA得分图、S-plot图及火山图

为避免使用一类统计方法带来的假阳性错误或模型过拟合,通过单变量分析中的*t*检验(*p*<0.05)、变异倍数(FC>2或<0.5)和多变量分析中变量投影重要性(VIP值)>1相结合的方法确定差异代谢物。

如表1所示,与空白对照组相比,环磷酰胺模型组上调了32种差异代谢物,下调了28种差异代谢物。激素替代治疗是为女性因卵巢功能衰退影响健康问题采用的临床医疗措施,结合雌激素单独^[17]^或配合其他药物一起使用^[18]^在缓解雌激素缺乏和治疗卵巢早衰方面具有不错的临床效果。实验中雌激素组与环磷酰胺模型组相比,上调了47种差异代谢物,下调了29种差异代谢物,与空白对照组相比,回调了34种差异代谢物。低浓度松花粉组与环磷酰胺模型组相比,上调了34种差异代谢物,下调了30种差异代谢物,回调了47种差异代谢物;中浓度松花粉组与环磷酰胺模型组相比,上调了32种差异代谢物,下调了37种差异代谢物,回调了38种差异代谢物;高浓度松花粉组与环磷酰胺模型组相比,上调了34种差异代谢物,下调了24种差异代谢物,回调了34种差异代谢物。空白对照组、雌激素组及松花粉干预组与环磷酰胺模型组的差异代谢物的韦恩图如图5所示,可以看出,相对结合雌激素的使用,松花粉可以在不造成更多代谢物改变的前提下,在回调环磷酰胺对肝脏代谢物改变方面有与雌激素相当甚至更好的表现,并且松花粉与雌激素都可以对2-羟基喹啉和3-吲哚丙酸等23种代谢物向空白对照组代谢物水平调节。

**表1:** 各组大鼠体内差异代谢物的变化

No.	Metabolite	MG vs CG	MG vs EG	MG vs PG. L	MG vs PG. M	MG vs PG. H
1	2-hydroxyquinoline	↑	↑	↑	↑	↑
2	3-indolepropionic acid	↑	↑	↑	↑	↑
3	4-guanidinobutanoic acid	↑	↑	↑	↑	↑
4	4-hydroxyquinoline		↑		↑	↑
5	decanoylcarnitine	↓	↓	↓	↓	↓
6	decenoylcarnitine	↓	↓	↓	↓	↓
7	dodecenoylcarnitine	↓	↓	↓	↓	↓
8	propionylcarnitine	↓	↓	↓	↓	↓
9	butyrylcarnitine		↓	↓		
10	2-octenoyl-L-carnitine	↓	↓	↓	↓	↓
11	allantoin		↑			↑
12	aminoadipic acid	↓	↓		↓	↓
13	3-amino-2-oxazolidinone		↑			
14	cinnamic acid		↑			
15	cytidine monophosphate	↓	↓	↓	↓	↓
16	cytosine	↑	↑	↑	↑	↑
17	D-(+)-octopine		↑	↑	↑	↑
18	deoxyadenosine monophosphate	↓	↓	↓	↓	↓
19	dimethylguanosine		↑			
20	estriol		↓			
21	gamma-aminobutyric acid		↑	↑	↑	
22	glycerophosphocholine	↑	↑	↑	↑	↑
23	glycodeoxycholic acid		↑		↑	↑
24	glycohyocholic acid	↑	↑		↑	↑
25	glycyl-glycine	↑	↑	↑	↑	↑
26	homo-L-arginine		↑		↑	↑
27	imidazoleacetic acid	↑	↑	↑	↑	↑
28	indole		↑			
29	indole-3-propionic acid		↑			↑
30	indolelactic acid		↑		↑	↑
31	indoline		↑			
32	L-cysteine		↑	↑		
33	lipoamide		↑		↑	↑
34	lysophosphatidylcholine 3∶1	↑	↑	↑		↑
35	nicotyrine		↑		↑	↑
36	O-butanoylcarnitine		↓			
37	ornithine		↑			
38	Phe-Phe		↑			↑
39	propionyl-carnitine	↓	↓	↓	↓	↓
40	purine		↑			↑
41	pyridoxal 5-phosphate	↓	↓	↓	↓	↓
42	ribothymidine		↑		↑	
43	trigonelline	↑	↑	↑	↑	↑
44	trimethylamine N-oxide	↑	↑	↑	↑	
45	uric acid		↓	↓	↓	↓
46	uridine 5-monophosphate	↑	↑	↑	↑	↑
47	2-aminooctanoic acid	↓				
48	adenosine monophosphate	↓		↓		↓
49	adenosine monophosphate	↓		↓		↓
50	cholic acid	↓		↓		
51	glycocholic	↑			↑	
52	glycerophosphocholine	↑		↑	↑	
53	inosinic acid	↓		↓	↓	↓
54	linolenic acid	↑				
55	Pro-Leu	↓		↓		
56	remerine	↑		↑		
No.	Metabolite	MG vs CG	MG vs EG	MG vs PG. L	MG vs PG. M	MG vs PG. H
57	uridine	↑		↑		
58	xanthosine	↑		↑		
59	2,3-diaminopropionic acid			↑		
60	decadienylcarnitine			↑		
61	acetyl-carnitine			↑		
62	L-acetylcarnitine			↑		
63	mannose 6-phosphate 2			↑		
64	Phe-Trp					↑
65	2-methylbutyroyl carnitine				↓	
66	3-pyridylacetic acid				↓	
67	isovalerylcarnitine				↓	
68	(S)-2-acetamidohexanoic acid				↓	
69	m-aminobenzoic acid				↓	
70	methylbutyroyl-carnitine				↓	
71	15,16-dihydroxy-9Z,12Z-octadecadienoic acid	↑	↑	↑	↑	↑
72	2-hydroxy-2-methylbutyric acid	↓	↓	↓	↓	↓
73	2-hydroxy-3-oxopropanoate		↓		↓	
74	3-phosphoglycerate	↓	↓	↓	↓	↓
75	3-α,20-α-dihydroxy-5-β-pregnane 3-glucuronide		↓	↑	↓	
76	4-hydroxybutanoic acid		↓		↓	
77	albiflorin	↓	↓	↓		↓
78	dehydroepiandrosterone sulfate/dihydrotestosterone		↓	↑	↓	
79	benzophenone-2	↑	↑	↑	↑	
80	biliverdin	↓	↓			
81	citrulline		↓		↓	↓
82	cortolone-3-glucuronide		↓		↓	
83	fatty acid 16∶1	↑	↑	↑	↑	
84	fatty acid 17∶1		↑		↑	
85	glycocholic acid	↑	↑		↑	↑
86	glycoursodeoxycholic acid		↑		↑	↑
87	haematommic acid		↓			↓
88	hippuric acid	↑	↑	↑	↑	↑
89	indolelactate		↑			↑
90	indoxyl sulfate		↑		↑	
91	inosine	↓	↓	↓	↓	
92	L-(-)-3-phenyllactic acid		↑			↑
93	L-tryptophan		↑			↑
94	paracetamol sulfate	↑	↑		↑	
95	phosphoenolpyruvate (PEP 1)	↓	↓	↓	↓	↓
96	taurodeoxycholic acid		↑		↑	↑
97	tetrahydroalstonine		↓			↓
98	trans-cinnamic acid		↑			↑
99	urate		↓		↓	↓
100	γ-glutamylphenylalanine		↑			
101	2-methylglutaric acid	↑				
102	2-naphthylamine	↓		↓	↓	↓
103	9,10-dihydroxy-12-octadecenoic acid	↓			↓	
104	cholic acid	↓		↓		
105	deoxycholic acid	↓		↓		
106	DL-p-hydroxyphenyl lactic acid	↓			↓	
107	D-ribose 5-phophate	↑		↑		
108	fructose 6-phosphate	↑		↑		
109	heptadecanoic acid	↑		↑		↑
110	hexadecanedioic acid	↓			↓	
111	N-acetyl-O-acetylneuraminate	↓		↓		
112	O-2545 (hydrochloride)	↓		↓		
No.	Metabolite	MG vs CG	MG vs EG	MG vs PG. L	MG vs PG. M	MG vs PG. H
113	p-cresol sulfate	↓		↓		
114	phosphoric acid	↓				↓
115	(S)-3-sulfolactate			↑		
116	7β,12α-dihydroxy-5α-cholan-24-oic acid			↓		
117	acetylenedicarboxylate			↓	↓	
118	deoxycholic acid 3-glucuronide			↓		
119	DL-glyceraldehyde 3-phosphate			↑		
120	fatty acid 20∶2			↑		
121	2-aminoethylphosphonic acid				↓	
122	fatty acid esters of hydroxy fatty acids 38∶3				↓	
123	1,6-bisphosphatase 1				↓	
124	erucic acid					↑

CG: the control group; MG: the model group; EG: the estrogen group; PG. L: low-dose pine pollen group; PG. M: medium-dose pine pollen group; PG. H: high-dose pine pollen group.

**图5:**
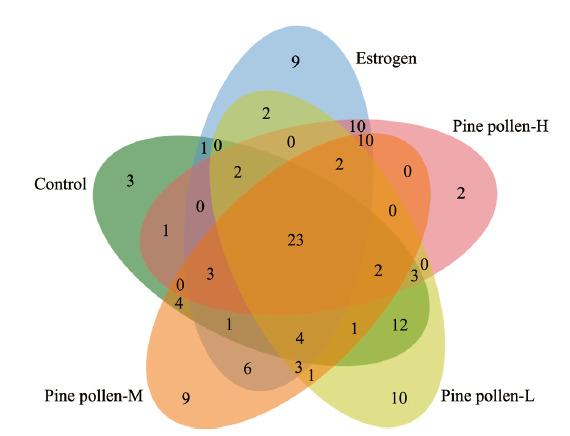
各分组差异代谢物的韦恩图

为了鉴定并可视化松花粉对卵巢早衰大鼠肝脏代谢的影响途径,使用MetaboAnalyst 5.0进行代谢组学途径的分析。结果显示,环磷酰胺通过*α*-亚麻酸代谢(影响值=0.3333, *p*值=0.20995)、维生素B6代谢(影响值=0.2549, *p*值=0.15035)、嘌呤代谢(影响值=0.18754, *p*值=0.005249)、赖氨酸降解(影响值=0.14085, *p*值=0.36557)和糖酵解/糖异生途径(影响值=0.1055, *p*值=0.39878)影响肝脏代谢。雌激素通过维生素B6代谢(影响值=0.2549, *p*值=0.20071)、赖氨酸降解(影响值=0.14085, *p*值=0.4651)、精氨酸和脯氨酸代谢(影响值=0.13448, *p*值=0.063537)、糖酵解/糖异生(影响值=0.1055, *p*值=0.47844)、半胱氨酸和蛋氨酸代谢(影响值=0.09592, *p*值=0.56315)等途径影响着被环磷酰胺诱导卵巢早衰大鼠肝脏的代谢紊乱。Palmisano等^[19]^报道了雌激素可以促进肝脏脂肪组织代谢,抑制脂肪生产和糖异生。同时,氨基酸与肝脏雌激素受体*α*(ERα)对生殖功能的调节在阐明闭经和不孕症的机制也具有重要意义^[20]^。松花粉则通过维生素B6代谢(影响值=0.2549, *p*值=0.091712)、嘌呤代谢(影响值=0.14578, *p*值=0.029752)、糖酵解/糖异生(影响值=0.1055, *p*值=0.24383)等途径影响被环磷酰胺诱导卵巢早衰大鼠肝脏的代谢紊乱。嘌呤是存在于生物体内的碱基,肝脏是其合成与代谢的重要场所,因而其代谢紊乱现象存在于酒精性肝病^[21]^等多种肝脏疾病患者中。玄参提取物^[22]^、川楝子^[23]^和栀子^[24]^等中药都被证明可以通过影响嘌呤代谢这一途径干预肝脏代谢。

选择松花粉与雌激素都参与有效回调的23种差异代谢物进行代谢通路分析,得到松花粉与雌激素影响肝代谢的相同途径是维生素B6的代谢(影响值=0.2549, *p*值=0.058236)。维生素B6是2-甲基-3-羟基吡啶酮的衍生物,是一种强抗氧化剂。最新研究显示维生素B6可通过抑制脂肪酸和胆固醇合成、促进脂肪酸分解和胆固醇转运改善肝脏脂质积累^[25]^。本研究中松花粉与雌激素相同的有效回调物质包含癸酰基肉碱等多种酰基肉碱。酰基肉碱是肉碱在将长链脂肪酸转运到线粒体过程中生成,当肉碱与酰基肉碱的含量比例失调时,可以认为体内发生代谢紊乱^[26]^,甚至疾病^[27]^。

## 3 结论

综上,松花粉可以有效回调环磷酰胺诱导卵巢早衰大鼠的肝脏代谢的变化,在不引起更多代谢物变化时,其回调效果与结合雌激素相当甚至更好,从代谢通路也可以看出雌激素在干预肝脏代谢时,存在精氨酸和脯氨酸代谢、半胱氨酸和蛋氨酸代谢两种新的代谢途径,而松花粉回调代谢物的通路属于环磷酰胺诱导的正常大鼠肝脏代谢紊乱时的代谢通路,未对机体新的代谢通路造成影响,且其影响途径主要是涉及维生素B6的代谢,主要体现在对酰基肉碱的调控上。该研究初步探明了松花粉对卵巢早衰大鼠肝脏代谢的干预机制,为卵巢早衰的机制及干预途径的探索以及松花粉的功能性应用开发提供了理论和实验基础。
